# Surface Treatment Effect on Shear Bond Strength between Lithium Disilicate Glass-Ceramic and Resin Cement

**DOI:** 10.1055/s-0041-1735908

**Published:** 2021-12-17

**Authors:** Siripan Simasetha, Awiruth Klaisiri, Tool Sriamporn, Kraisorn Sappayatosok, Niyom Thamrongananskul

**Affiliations:** 1 Department of Prosthodontics, Faculty of Dentistry, Chulalongkorn University, Bangkok, Thailand; 2 Division of Operative Dentistry, Faculty of Dentistry, Thammasat University, Pathumthani, Thailand; 3 Department of Prosthodontics, College of Dental Medicine, Rangsit University, Pathumthani, Thailand; 4 Department of Oral Diagnostic Science, College of Dental Medicine, Rangsit University, Pathumthani, Thailand

**Keywords:** lithium disilicate, resin cement, silane, shear bond strength

## Abstract

**Objective** The study aimed to evaluate the shear bond strength (SBS) of lithium disilicate glass-ceramic (LDGC) and resin cement (RC) using different surface treatments.

**Materials and Methods** LDGC blocks (Vintage LD Press) were prepared, etched with 4.5% hydrofluoric acid, and randomly divided into seven groups ( *n* = 10), depending on the surface treatments. The groups were divided as follows: 1) no surface treatment (control), 2) Silane Primer (KS), 3) Signum Ceramic Bond I (SGI), 4) Signum Ceramic Bond I/Signum Ceramic Bond II (SGI/SGII), 5) experimental silane (EXP), 6) experimental silane/Signum Ceramic Bond II (EXP/SGII), and 7) experimental silane/Adper Scotchbond Multi-purpose Adhesive (EXP/ADP). The specimens were cemented to resin composite blocks with resin cement and stored in water at 37 °C for 24 hours. The specimens underwent 5,000 thermal cycles and were subjected to the SBS test. Mode of failure was evaluated under the stereo microscope.

**Statistical Analysis** Data were analyzed with Welch ANOVA and Games-Howell post hoc tests (α = 0.05).

**Results** The highest mean SBS showed in group EXP/ADP (45.49 ± 3.37 MPa); however, this was not significantly different from group EXP/SGII (41.38 ± 2.17 MPa) ( *p* ≥ 0.05). The lowest SBS was shown in the control group (18.36 ± 0.69 MPa). This was not significantly different from group KS (20.17 ± 1.10 MPa) ( *p* ≥ 0.05).

**Conclusions** The different surface treatments significantly affected the SBS value between LDGC and RC. The application of pure silane coupling agent with or without the application of an adhesive improved the SBS value and bond quality.

## Introduction

As dental materials have improved, patients have tended to demand esthetics and metal-free restorations. This demand has caused the clinical application of all-ceramic restoration to become widely used. 1 All-ceramic materials can display aesthetics to mimic the tooth color. The successful clinical application of all-ceramic materials depends on the clinicians' ability to develop the appropriate treatment plans. They must then carefully choose the appropriate material and cementation protocol to fulfill the patients' needs and expectations.

Lithium disilicate restorations are usually monolithic, in which the full contour of the prosthesis is fabricated from a homogeneous single material. It has translucency, opalescence, and light diffusion that can be stained, glazed, or cut back to layer veneering porcelain to enhancing incisal characterization. Lithium disilicate can be used for inlays, onlays, three-quarter crowns, full-coverage crowns, and short-span bridges in the anterior region. 2

The clinical service outcome of lithium disilicate restoration largely depends on the resin-ceramic bond. Strong and substantial resin bonding increases retention, 3 improves marginal adaptation, 4 reduces bacterial microleakage, 5 and improves fracture resistance. 6 The resin-ceramic bond can be generated through micromechanical retention, chemical bonding to a silica-based ceramic surface, and surface wettability. 7 To gain micromechanical retention, the surface can be prepared by airborne particle abrasion and/or etching with hydrofluoric acid. Airborne particle abrasion is not suggested as a result of a significant reduction in the flexural strength, 8 and does not give a favorable bond strength to lithium disilicate glass-ceramic (LDGC). 7 Hydrofluoric acid etching dissolves the glass phase from the matrix and creates microporosity and increases the surface areas. 9 Chemical bonding between the resin-ceramic surfaces can be accomplished by using a silane coupling agent. The silane coupling agent is a bifunctional molecule that encourages adhesion through the covalent bonds with hydroxyl (OH) groups on the ceramic surface. One functional group can react with the inorganic ceramic surface and the other methacrylate group is capable of reacting with an organic resin matrix. Silane bonds to Si-OH on a ceramic surface by a condensation reaction. The methyl methacrylate double bonds provide bonding to the adhesive. The application of unfilled resin before cementation with resin cement (RC) will enhance infiltration into the superficial irregularities of etched LDGC, resulting in increased bond strength. 10 Furthermore, the RC plays an important role in the bond to high-crystalline content ceramics. Adhesion between dental ceramics and resin cement is the result of a physicochemical interaction across the interface between resin and ceramic.

Therefore, the purpose of this study was to evaluate the shear bond strength (SBS) of LDGC and RC using different surface treatments. The null hypotheses were as follows: 1) there would be no difference to be found in SBS between LDGC and RC using different silane surface treatments and 2) there would be no difference to be found in SBS between LDGC and RC using different silanes followed by different adhesives.

## Materials and Methods

### Lithium Disilicate Specimen Preparation

LDGC (Vintage LD Press ceramic ingot, Shofu Inc., Kyoto, Japan) was pressed into the circular mold with 5 mm diameter and 3 mm length in an automatic press furnace (PANAMAT 640/620, DEKEMA Dental-Keramikofen GmBH, Freilassing, Germany). The specimens were observed under stereo microscope (SZ61, Olympus Corporation, Tokyo, Japan) at 40x magnification to evaluate ceramic surface defects. The defective ones were excluded. The specimens were randomly divided into 7 groups ( *n* = 10). Each specimen was embedded in a PVC block size 20 mm diameter and 25 mm length, using self-curing acrylic resin (Ortho-Jet, Lang, Illinois, U.S.A.). The upper surface was polished to a flat surface with wet polishing using 320, 600, and 800 grit silicon carbide abrasive paper (3M Wetordry abrasive sheet, 3M-ESPE St. Paul, Minnesota, U.S.A.) and a polishing machine (Nano 2000 grinder-polisher with FEMTO 1000 polishing head, Pace Technologies, Arizona, U.S.A.) with a load of 2 kg/cm ^2^ . The silicon carbide paper was rotated at a speed of 100 rotations per minute (rpm) in antirotation movement, and the specimens were rotated in rotation movement. The polishing cycle is 2 minutes. The silicon carbide paper was changed when the polish cycle ended. All the specimens were immersed in an ultrasonic cleaner (VGT-1990 QTD, Guangzhou, Guangdong, China) for 10 minutes to remove debris. Again, the specimens were observed under Stereo Microscope (SZ61, Olympus Corp, Tokyo, Japan) at 40x magnification to evaluate surface defects after the polishing cycle. The defective specimens were excluded. Then, the specimens were subsequently etched with 4.5% hydrofluoric acid (IPS Ceramic Etching-gel, Ivoclar Vivadent, Schaan, Liechtenstein) for 20 seconds, rinsed with water, and dried with air blow. Finally, they were again immersed in the ultrasonic cleaner for 10 minutes. The surface treatment of each experimental group is described in Table 1 . The adhesive tape (Scotch 3M Tape, 3M-ESPE St. Paul, Minnesota, U.S.A.) with 2.38 mm diameter (ISO 29022:2013) 11 and 50 µm thickness (ISO 4049:2009) 12 was firmly attached to the upper surface of Vintage LD Press specimen to define the area of bonding, and control the film thickness of the RC. The materials used in this study are shown in Table 2 .

**Table 1:** Codes and surface treatment of each experimental groups

Groups	Codes	Surface treatments
1	Control	No silane treatment
2	KS	Surface treatment with KS. The micropipette was used to take 3 microliters of KS and placed on the etched ceramic surface then applied with a disposable micro applicator as a single film and left untouched for 60 seconds.
3	SGI	Surface treatment with SGI. The micropipette was used to take 3 microliters of SGI and placed on the etched ceramic surface then applied with a disposable micro applicator as a single film, left untouched for 10 seconds, and allowed to dry.
4	SGI/SGII	Surface treatment with SGI as previously described in group 3 and followed by application of SGII. The micropipette was used to take 3 microliters of SGII on the silanized ceramic surface as a thin layer and rubbed it for 30 seconds. Any excess will be removed with a new disposable micro applicator.
5	EXP	Surface treatment with EXP. The micropipette was used to take 3 microliters of EXP and placed on the etched ceramic surface then applied with a disposable micro applicator as a single film, left untouched for 60 seconds, and allowed to dry.
6	EXP/SGII	Surface treatment with EXP as described in group 5, followed by SGII as described in group 4.
7	EXP/ADP	Surface treatment with EXP as described in group 5, followed by ADP. The micropipette was used to take 3 microliters of ADP and applied on the silanized ceramic surface then rubbed with a disposable micro applicator as a single film, left untouched for 60 seconds, and allowed to dry. Any excess will be removed with a new disposable micro applicator.

Abbreviations: ADP, Adper Scotchbond Multi-purpose Adhesive; EXP, experimental silane; KS, Kerr Silane Primer; SGI, Signum Ceramic Bond I; SGII, Signum Ceramic Bond II.

**Table 2:** Trade names, manufacturers, compositions of materials, and lot numbers used in this study

Trade names	Manufacturers	Compositions	Lot number
Vintage LD Press	Shofu Inc., Kyoto, Japan	Lithium disilicate-based ceramic	021601
IPS-Ceramic Etching Gel	Ivoclar Vivadent, Schaan, Liechtenstein.	Aqueous solution of hydrofluoric acid	Y34242
Silane Primer	Kerr Corp., Orange, CA, U.S.A.	gamma-MPS, BisEMA, TEGDMA, and ethanol	6825763
Signum Ceramic Bond I + II	Kulzer, Hanau, Germany.	Signum Ceramic Bond I: Isopropanol, acetone, silane, acids, monomer, initiators, and stabilizers	K010112
		Signum Ceramic Bond II: Silane, initiators, stabilizers, monomers, and silicic acid	K010711
Experimental silane	Sigma-Aldrich, St. Louis, MO, U.S.A.	gamma-MPS, distilled water, and acetic acid, and alcohol	SHBJ3136
Adper Scotchbond Multi-purpose Adhesive	3M ESPE, St. Paul, MN, U.S.A.	Bis-GMA, HEMA, and initiators	N979519
RelyX U200	3M ESPE, St. Paul, MN, U.S.A.	Base: methacrylate monomers containing phosphoric acid groups, methacrylate monomers, silanated fillers, initiator components, stabilizers, and rheological additives Catalyst: methacrylate monomers, alkaline (basic) fillers, silanated fillers, initiator components, stabilizers, rheological additives, and pigments	4819681
Filtek Z350 XT Universal Restorative	3M ESPE, St. Paul, MN, U.S.A.	Organic matrix: BisGMA, BisEMA, UDMA, and TEGDMA Inorganic particle: nonagglomerated nanoparticles of silica and nanoagglomerates formed of zirconium/silica particles	N912324

### Preparation of Experimental Silane

The experimental silane used in this study was prepared as per a previous study 13 through a mixing of a ratio of 70% ethanol and 30% distilled water in a glass container. The pH of the solution was changed to the range of 4.5 to 5.5 using acetic acid and measured for accuracy using a digital pH meter (Orion 420a pH, Thermo Electron Corp, Massachusetts, U.S.A.). The solution was then moved to a new plastic container, where it was then mixed with a silane coupling agent: 3-trimethoxysilyl propyl methacrylate (gamma-MPS) (Sigma-Aldrich, Missouri, U.S.A.). The mixing process entailed slowly adding the agent, using a stirring procedure to produce a 2% concentrated solution. It was then left for 60 minutes with no actions to allow hydrolysis to take place and form the final silane mixture. At that point, a magnetic stirrer and bar (Hotplate stirrer UC152, Stuart Scientific, Staffordshire, U.K.) were used to gently mix the solution for 10 minutes.

### Resin Composite Block Preparation

The resin composite blocks (Filtek Z350XT,3M ESPE, St. Paul, Minnesota, U.S.A) were fabricated from putty silicone mold with 3 mm diameter and 3 mm length, using the light-curing unit (Bluephase N, Ivoclar Vivadent, Schaan, Liechtenstein) with the intensity of 1200 mW/cm, 2 as per the manufacturer's instruction.

### Resin Composite Block Cementation

**Fig. 1:**
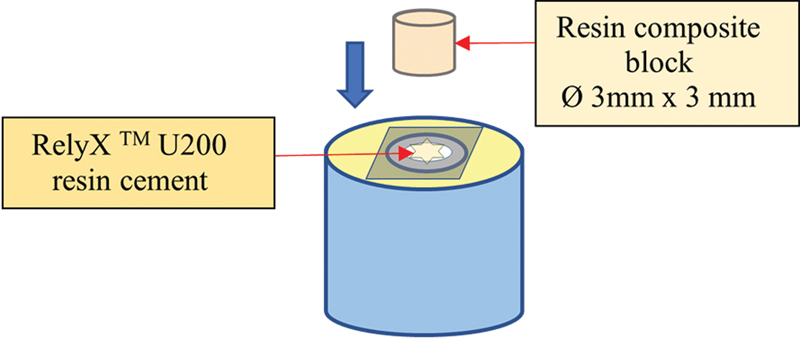
Resin composite block cementation to the bonding area.

The self-adhesive RC (RelyX U200, 3M ESPE, St. Paul, Minnesota, U.S.A.) was cemented with the Vintage LD Press ( Fig. 1 ) as per the manufacturer's instruction by mixing the base paste and catalyst paste on the mixing pad and then placing the composite block on the treated Vintage LD Press surface under a constant weight of 1,000 g. Remove the excess cement with a new disposable micro applicator (Cotisen micro applicator dispenser, Huanghua premise dental, Huanghua, Hebei, China). The light-curing unit was used to apply on four joining surfaces for 40 seconds per joining surface. After that, the specimens were placed in distilled water and stored in the Incubator (Contherm 160 M, Contherm Scientific Ltd., Wellington, New Zealand) under 37 °C for 24 hours. The specimens underwent 5,000 thermal cycles (Thermo Cycling Unit, King Mongkut's Institute of Technology Ladkrabang, Bangkok, Thailand) with a 30-second dwell time and a 5-second transfer time between 5 and 55 °C and subjected to the SBS test.

### Shear Bond Strength Test

All of the specimens were mounted in the universal testing machine (EZ-S 500N, Shimadzu Corporation, Kyoto, Japan). The SBS was tested using the notched-edge shearing blade. The notched-edge shearing blade was placed parallel to the bonding site. The crosshead speed is 1.0 mm per minute. The SBS in megapascal (MPa) was determined from the maximum load prior to the bond failure (N) divided by the bonding area (mm ^2^ ) between RC and LDGC. After this, the fractured parts were evaluated with stereo microscope (SZ 61, Olympus, Tokyo, Japan) at 40x magnification. The mode of failure was adapted from Matinlinna and Lassila 14 and categorized into three types. When less than 40% of the RC could be observed on the surface of LDGC, it was categorized as an adhesive failure (AF), meaning that there is no bond between LDGC and RC. When at least 60% of the RC could be observed on the surface of LDGC, it was categorized as a cohesive failure (CF), meaning that failure occurs in the RC. When more than 40% but less than 60% of the RC could be observed on the surface of LDGC, it was categorized as a mixed failure (MF), meaning that it has both adhesive and cohesive failures.

The percentage of area in mode of failure was measured using ImageJ software and then calculated using the marked area of RC on the bonded ceramic surface, divided by the total bonded area and multiplied by 100, as in the following example.

- MF was categorized when more than 40% but less than 60% of the RC could be observed on the surface of LDGC.

**Fig. 2:**
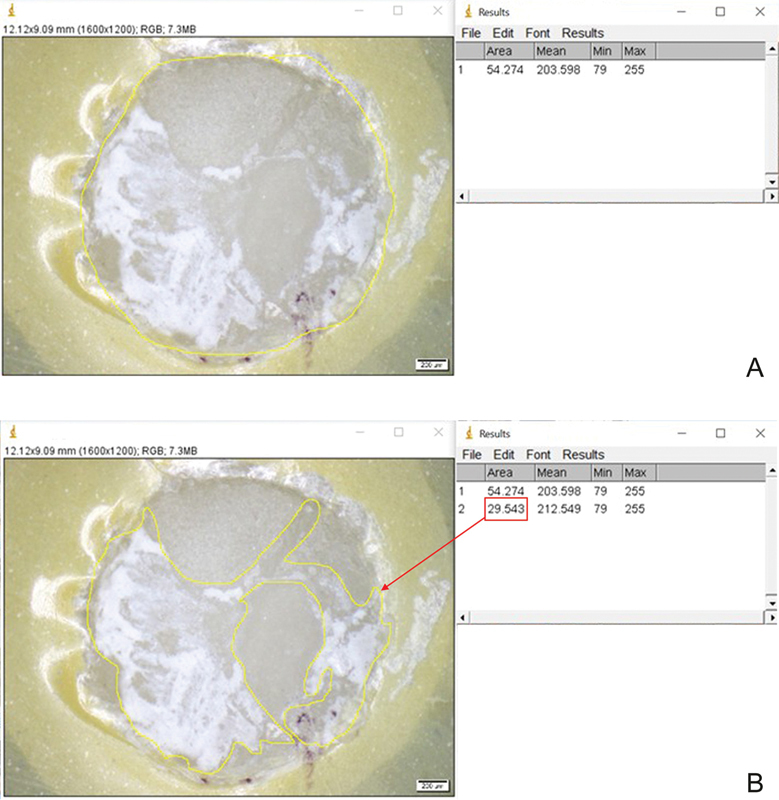
The percentage of area in mixed failure (MF) was determined using ImageJ software.

An example of the percentage of area in MF was measured using ImageJ software. The total bonded area measured was 54.27 ( Fig. 2A ), and the area of RC was 29.54 ( Fig. 2B ). Therefore, the percentage of area in mode of failure was calculated from the area of RC, divided by the total bonded area and multiplied by 100, which was 54.41%.

**Fig. 3:**
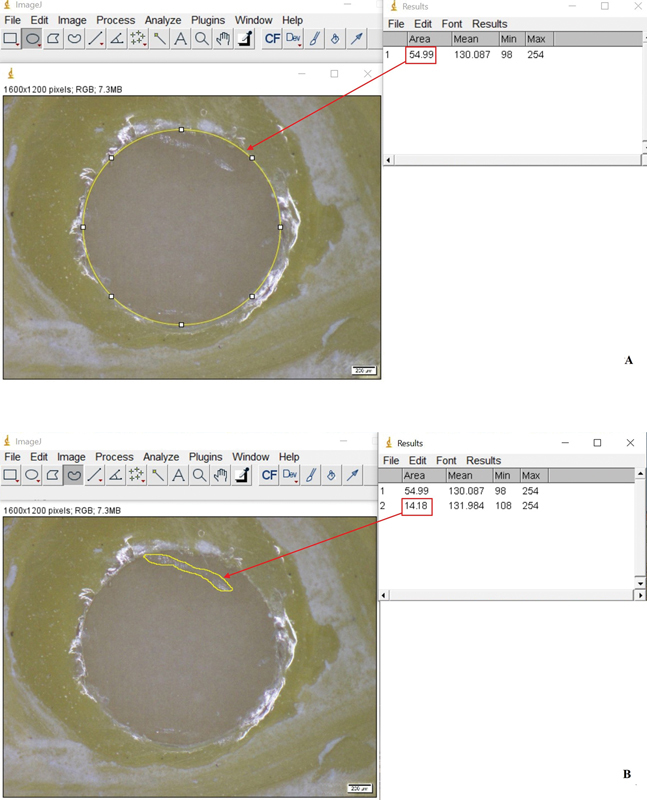
The percentage of area in adhesive failure (AF) was determined using ImageJ software.

**Fig. 4:**
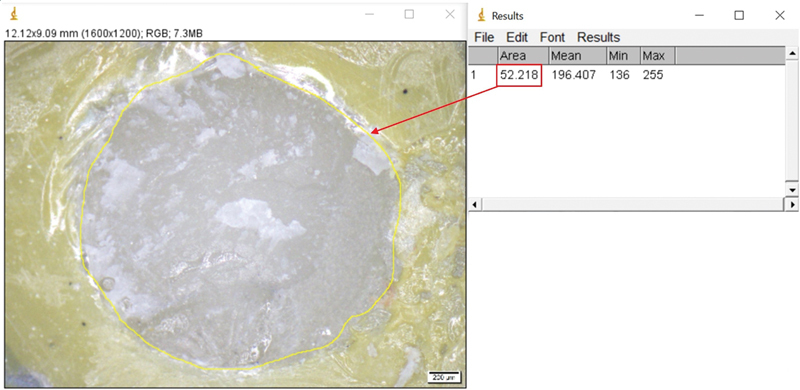
The percentage of area in cohesive failure (CF) was determined using ImageJ software.

- AF was categorized when less than 40% of the RC could be observed on the surface of LDGC. An example of the percentage of area in AF was measured using ImageJ software. The total bonded area measured was 54.99 ( Fig. 3A ), and the area of RC was 14.18 ( Fig. 3B ). The percentage of area in mode of failure was calculated from the area of RC, divided by the total bonded area and multiplied by 100, which was 25.7%.
- CF was categorized when at least 60% of the RC could be observed on the surface of LDGC. An example of percentage of area in CF was measured using ImageJ software. The total bonded area measured was 52.22 ( Fig. 4 ), and the area of RC was on the entire bonded surface, which was 52.22. The percentage of area in mode of failure was calculated from the area of RC, divided by the total bonded area and multiplied by 100, which was 100%.

### Statistical Analysis

The data was analyzed by IBM SPSS Statistic for Windows version 22.0. Welch ANOVA and Games-Howell post hoc multiple comparisons tests were used to analyze the difference among groups. All *p* -values < 0.05 were considered statistically significant.

## Results

According to the statistical analysis results using Welch ANOVA, the SBS was significantly affected by the different silane surface treatments' application. The mean SBS obtained from silane surface-treated groups is shown in MPa and the mode of failure after 5,000 thermocycling is shown in Table 3 . The lowest SBS was shown in a control group, which was not significantly different from group 2 ( *p* ≥ 0.05). However, the SBS of groups 1 and 2 were significantly different from groups 3, 4, 5, 6, and 7 ( *p* < 0.05). The SBS of group 4 was significantly higher than group 3 ( *p* < 0.05). The SBS of group 4 and group 5 were not significantly different ( *p* ≥ 0.05). Meanwhile, the SBS of group 4 was significantly different from groups 6 and group 7 ( *p* < 0.05). Group 7 exhibited the highest SBS but was not significantly different from group 6 ( *p* ≥ 0.05).

**Table 3:** Means and SD of SBS obtained from each respective silane surface treatment groups in MPa and mode of failure

Groups	Surface treatments	Mean ± SD (MPa)	Mode of failure (AF/CF/MF)
1	No surface treatment (control)	18.36 ± 0.69 ^a^	10/0/0
2	KS	20.17 ± 1.10 ^a^	9/0/1
3	SGI	25.16 ± 1.35 ^d^	3/4/3
4	SGI/SGII	30.03 ± 2.80 ^b^	1/5/4
5	EXP	32.52 ± 1.32 ^b^	1/6/3
6	EXP/SGII	41.38 ± 2.17 ^c^	0/6/4
7	EXP/ADP	45.49 ± 3.37 ^c^	0/7/3

Abbreviations: ADP, Adper Scotchbond Multi-purpose Adhesive; AF, adhesive failure; CF, cohesive failure; EXP, experimental silane; KS, Kerr Silane Primer; MF, mixed failure; SBS, shear bond strength; SD, standard deviation; SG I, Signum Ceramic Bond I; SG II, Signum Ceramic Bond II.

The same superscript indicates no significant difference ( *p* ≥ 0.05).

**Fig. 5:**
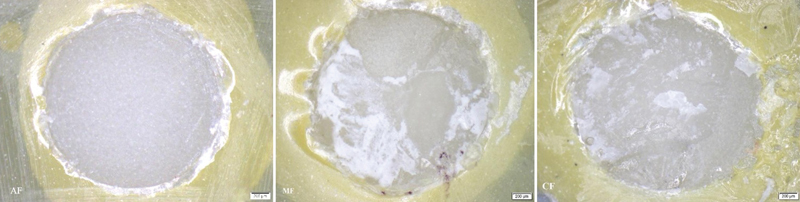
The stereo microscopic images of adhesive failure (AF) of group 1, mixed failure (MF) of group 6, and cohesive failure (CF) of group 7.

The results of the mode of failure evaluation under the stereo microscope at the magnification of 40x have shown that AF was highly exhibited in group 1 (100%) and group 2 (90%). This was when less than 40% of the RC could be observed on the surface of LDGC, meaning that there is no bond between LDGC and RC. However, the AF was not exhibited in groups 6 and group 7. CF was highly exhibited in group 7 (70%), group 6 (60%), and group 5 (60%). This was when 60% of the RC could be observed on the surface of LDGC, meaning that failure occurs in the RC. MF was exhibited approximately 30 to 40% in groups 3, 4, 5, 6, and 7. This was when more than 40% but less than 60% of the RC could be observed on the surface of LDGC, meaning that it has both AF and CF. The samples of stereo microscope images of AF, MF, and CF are shown in Fig. 5 .

## Discussion

This study evaluated the SBS of LDGC and RC using different surface treatments. Results of this study found there were significant differences of SBS between LDGC and RC using different silanes surface treatments. The study also found there were significant differences in SBS between LDGC and RC using different silanes followed by different adhesives. Therefore, the null hypotheses were rejected.

In this study, all of the specimens were etched with 4.5% hydrofluoric acid to simulate the clinical practice. It is recommended the silica-based ceramic have a proper surface treatment. The recommended treatment involves etching the ceramic internal surface with hydrofluoric acid and applying silane coupling agent to create micromechanical retention. 15 16 Acid-etching creates microporosity, and increases the surface area 17 and a high-free energy surface state. 9 This will decrease the contact angle between the LDGC and RC. Acid-etching increases surface wettability for silane coupling agents, resulting in bond strength and bond durability improvement. All of the specimens underwent the thermal cycling test for 5,000 cycles. Thermocycling is used to predict clinical service when there is an initial seal between materials under pressure, so that the test will be clinically representative. 18

The SBS of group 2 was not different from the control group. Group 2: Silane Primer is a resin-containing silane coupling agent which was developed to reduce clinical surface pretreatment steps and expected to bond with resin in the resin luting cement. Chen et al study on Silane Primer application on etched lithium disilicate surface contact angle was not significantly different from untreated etched lithium disilicate surface angle. The similar contact angle of unetched and etched lithium disilicate glass-ceramic suggested that the chemical bond between LDGC surface and silane did not occur or only slightly occurred. There are many factors affecting bond quality. Surface wettability and surface energy are some of the factors. The additive-like resin may impede condensation reaction of the silane coupling agent, causing low-bond strength, low-contact angle, 19 and does not enhance bond strength between RC and LDGC. 20 The condensation reaction formed stabilized siloxane and released water. The addition of resin in the silane coupling agent may delay water vaporization. 19 Adding resin in silane coupling agent causes instability of the chemical substrate and might interfere with the condensation reaction of the hydroxyl group on the lithium disilicate surface and silanol. 19 The pH value of Silane Primer is at 7.3 which has lower acidity than an appropriate hydrolysis pH which occurs at 4. 21 Furthermore, Dimitriadi et al 22 stated that Silane Primer showed slight silanol activity, which was confirmed by the similar siloxane (Si-O-Si) peak between the etched ceramic and polished ceramic surfaces. These might be the reasons that Silane Primer has lower bond strength than the other groups. Meanwhile, in group 1, the control gained some SBS from the hydrofluoric acid etching on LDGC, creating microporosity and hence increasing in surface area and surface energy. 10 15 23 24

Signum Ceramic Bond I (SGI) was considered as a silane coupling agent. 25 26 Meanwhile, Signum Ceramic Bond II (SGII) was considered as an adhesive. 26 It could be implied that group 4 (SGI/SGII) had higher SBS than group 3 (SGI) because of the applied adhesive. Some studies recommended the use of an unfilled resin as an optional procedure to enhance surface wettability, reduce etched surface irregularities, and reinforce etched lithium disilicate surface. This resulted in gaining a high bond strength. 27 28 The bond quality was improved in group 4 and a higher MF was observed than in group 3. Exhibition of MF and reduction of adhesive failure in group 4 referred to the bond quality improvement. 29

Group 5: application of an experimental silane (EXP) showed significantly higher bond strength than group 3 (SGI). This may be due to the different types of solvent used, the concentration of the silane, and silane molecular structure in the manufacturing process. The hydrophilicity of solvent affects the hydrolysis rate. 30 EXP solvent was ethanol, which has lower hydrophilicity than propanol. Isopropanol and acetone were used as solvents in SGI. Furthermore, the variation of liquid solvent affects the surface tension. The lower surface tension solvents tend to have a favorable wettability. The EXP was mixed at a pH of 4.6 and the pH value of SGI was 4.5, which both correspond to the optimum hydrolysis rate of silanol occurring at a pH value of 4. 21 Thus, the hydrolysis pH factor was not considered among these groups. EXP and SGI were considered as conventional prehydrolyzed silanes, which have a more favorable silanol activity than the universal adhesive. 22 This caused group 2 (KS) to have lower bond strength than group 5 (EXP) and group 3 (SGI).

Meanwhile, group 4 (SGI/SGII) and group 5 (EXP) were not different in SBS. This may be due to variations of solvents used in each silane group. Ethanol was mainly used as solvent, and silane dissolves better in ethanol than water. Moreover, silane molecular structure, silane concentration, temperature, and humidity may affect silane hydrolysis. 30

SBS of Group 6 (EXP/SGII) was not different from the application of EXP, followed by Adper Scotchbond Multi-purpose Adhesive (ADP). ADP contained no silane coupling agent. It was composed of Bis-GMA and hydroxyethyl methacrylate (HEMA), tertiary amine, and initiator. This adhesive was used in a three-step total-etch adhesive system. Meanwhile, the SGII composition showed that there was a presence of silane coupling agent, but the amount of silane coupling was not shown. It could be implied the amount and concentration of silane coupling agent in the bonding agent might have been insufficient, causing an adverse effect on the bonding agent. However, group 6 and group 7 exhibited more CF than the other groups, and no AF was observed. This showed a better bond quality when using an adhesive as an optional surface treatment. When comparing the commercial silanes and experimental silane with or without an adhesive application, high SBS values and CFs were found in the experimental groups. This occurred because the experimental silane groups reached an optimal pH silane hydrolysis rate, which occurred at a pH value of 4. The pH value is the main factor affecting silane hydrolysis. The experimental silane used ethanol as a solvent, because silane is more easily dissolved in ethanol. It also has a higher hydrophilicity than propanol, which makes it tend to have a higher hydrolysis rate. 30 These are the reasons experimental silane exhibited high bond strength and bond quality. The results of this study corresponded with the previous study. 27 28 The results of the study showed the bond strength value correlated with the mode of failure. The high SBS value groups tended to have more mixed and cohesive failures than the ones that had a lower SBS value which tended to have more AF.

There are some limitations/possible limitations in this study; This study was an *in vitro* study. It could not fully simulate a cyclic load in the oral condition completely. Furthermore, contact angles of each silane coupling agent were not demonstrated. This might affect the surface wettability of silane. Therefore, further studies may require using various types of additives in the silane coupling agents to evaluate surface wettability and bond strength.

## Conclusions

The different types of surface treatments significantly affect the SBS value between LDGC and RC. Application of conventional prehydrolyzed silane with or without the application of an adhesive improves the SBS value and bond quality. The presence of MFs and CFs indicate bond quality improvement.
